# Supplementary material for: Engineering mammalian cells for detection and treatment of cardiac injury
Source: Mol Syst Biol. 2025 Oct 10;22(1):88–101. doi: 10.1038/s44320-025-00161-x (PMC12759069; doi:10.1038/s44320-025-00161-x)
Supplement: Supplementary file 1 — Appendix [file 44320_2025_161_MOESM1_ESM.pdf]

Appendix for Engineering mammalian cells for detection and treatment of cardiac injury

Appendix Figure S1	Comparative analysis of TropR expression levels by flow cytometry	P2
Appendix Figure S2	TropR variants engineered with different intracellular signaling domains	P3
Appendix Figure S3	Potential impact of TropR on endogenous signaling pathways	P4
Appendix Figure S4	Estimation of the TNK efficacy window using ex vivo thrombolysis assays	P5
Appendix Figure S5	CardioProtect sensitivity to short cTnI exposure times	P6
Appendix Figure S6	Doxycycline-repressible TNK production by encapsulated CardioProtect	P6
Appendix Figure S7	Temporal evolution of the ex vivo blood culture assay	P7
Appendix Table S1	Plasmids designed and used in this study	P8

Supplementary Figures

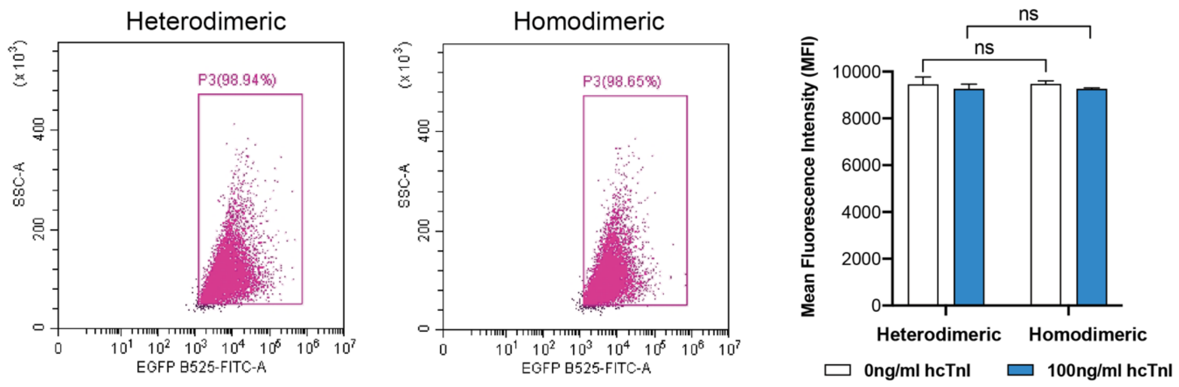

Appendix Fig. S1. Comparative analysis of TropR expression levels by flow cytometry. HEK-293 cells expressing either heterodimeric (co-transfection of pYX941 and pYX942, 50 ng each) or homodimeric enhanced green fluorescent protein (EGFP)-tagged TropR (transfection of pYX943, 200 ng) were subjected to flow-cytometric analysis of green fluorescence, indicative of receptor expression levels, after 24 h. Representative plots for n = 3 independent experiments are shown.

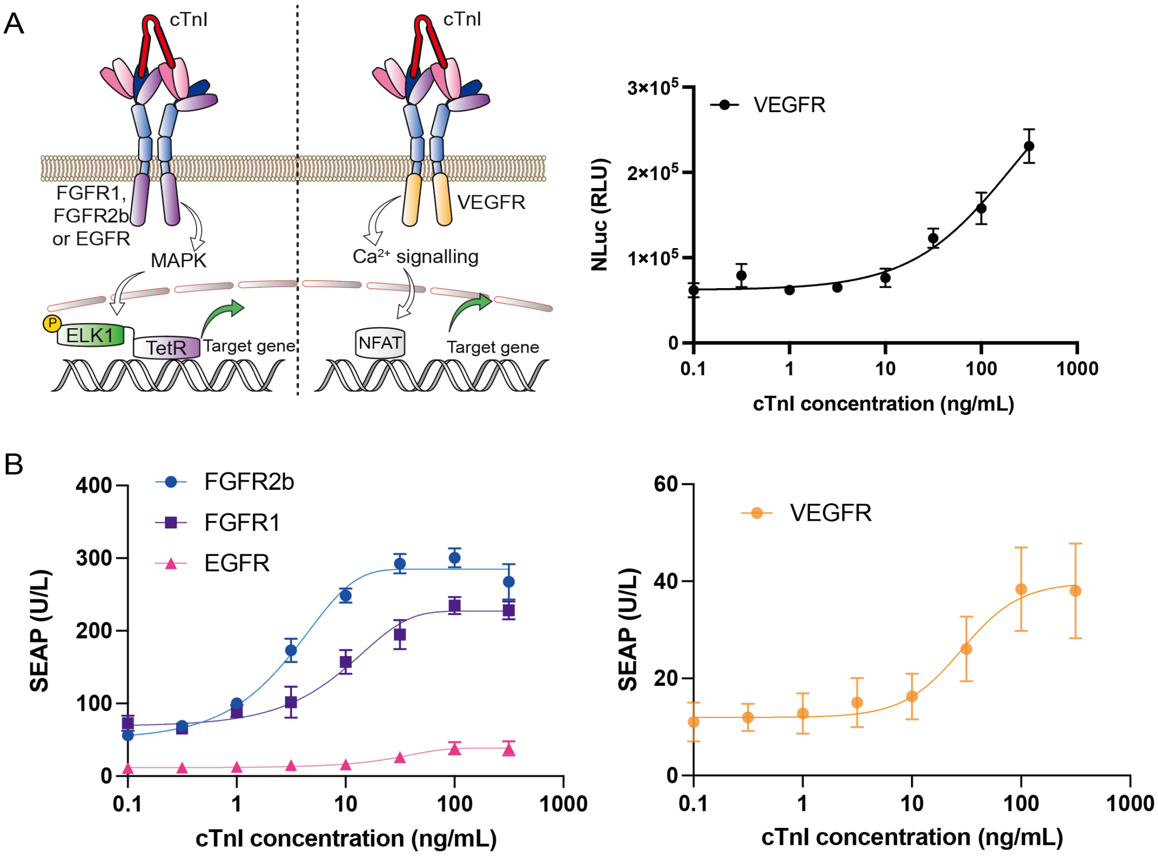

Appendix Fig. S2. TropR variants engineered with different intracellular signaling domains (related to Fig. 3A). (A) Gene expression control by cTnI-triggered calcium signaling. HEK-293 cells were co-transfected with a synthetic NFAT-specific promoter secreted *Oplophorus gracilirostris* luciferase containing a IgK-derived signal peptide (NanoLuc) expression vector (pYX415) and a TropR expression vector containing a VEGFR-derived intracellular signaling domain (pLeo1171) before cultivation in culture medium containing different concentrations of human cTnI. NanoLuc levels in culture supernatants were quantified at 24 h after cTnI stimulation. **(B) TropR-dependent human placental secreted alkaline phosphatase (SEAP) expression.** (left) HEK-293 cells were co-transfected with a constitutive TetR-Elk1-expression vector (Mkp37), a synthetic TetR-specific SEAP expression vector (pMF111) and a TropR expression vector containing FGFR2b- (pLeo1164), FGFR1- (pLeo1061) or EGFR-derived intracellular signaling domains (pLeo1165), and then cultivated in culture medium containing different concentrations of human cTnI. (right) HEK-293 cells were co-transfected with a synthetic NFAT-specific promoter SEAP expression vector (pYL1) and a TropR expression vector containing a VEGFR-derived intracellular signaling domain (pLeo1171) before cultivation in culture medium containing different concentrations of human cTnI. SEAP levels in culture supernatants were quantified at 24 h after cTnI stimulation. All data are presented as the mean ± SD, n = 3 independent experiments.

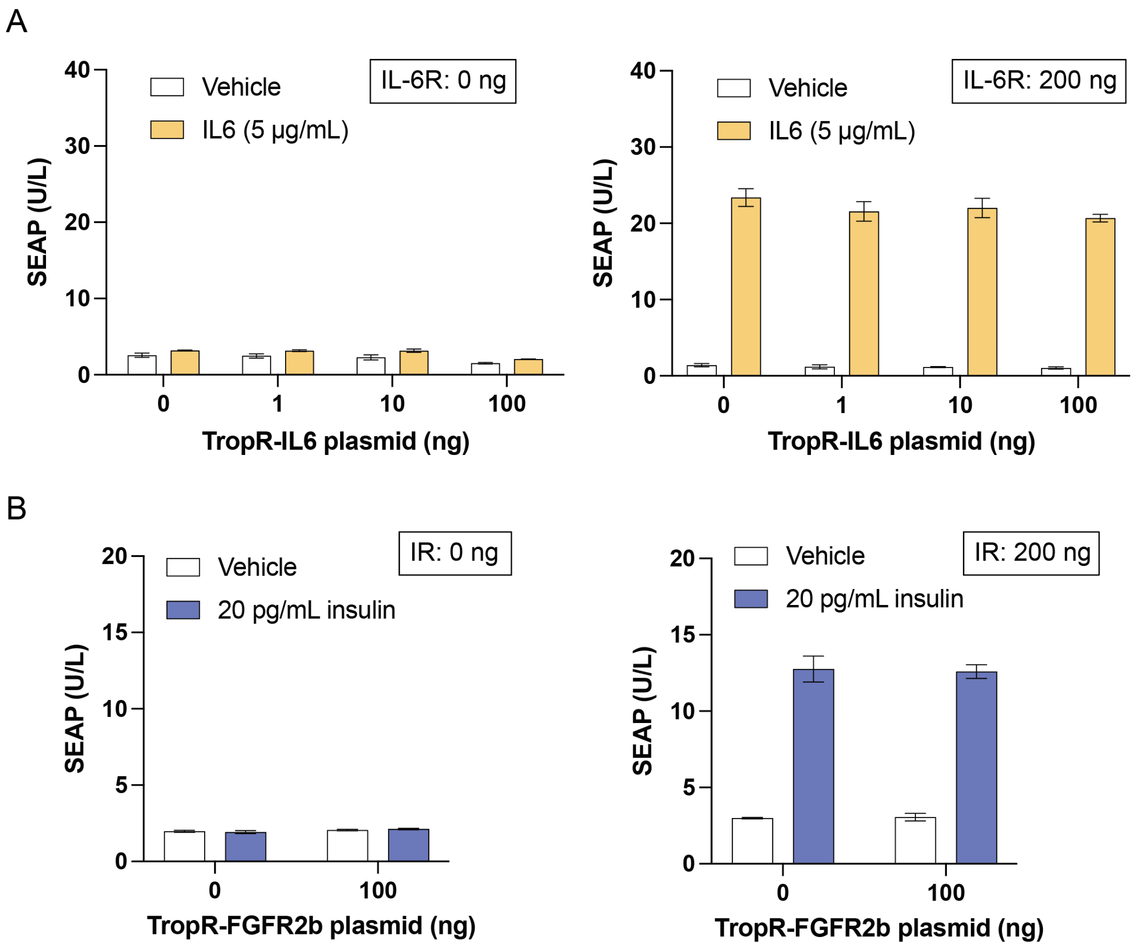

Appendix Fig. S3. Potential impact of TropR on endogenous signaling pathways. (A) Impact of TropR on endogenous signal transducer and activator of transcription isoform 3 (STAT3) signaling. HEK-293 cells were co-transfected with a synthetic STAT3-specific SEAP expression vector (pLZ284), a constitutive expression vector for human interleukin 6 receptor (pIL-6R or pcDNA3.1(+) as a negative control) and different amounts of a constitutive $\text{TropR}_{\text{IL-6R}}$ expression vector (pLeo1060). SEAP levels in culture supernatants were measured at 24 h after stimulation with recombinant human IL-6 (5 µg/mL). **(B) Impact of TropR on endogenous MAPK signaling.** HEK-293 cells were co-transfected with a TetR-specific SEAP expression vector (pMF111), constitutive expression vectors for TetR-Elk1 (Mkp37) and human insulin receptor (pIR or pcDNA3.1(+) as a negative control) and different amounts of a constitutive $\text{Trop}_{\text{FGFR2b}}$ expression vector (pLeo1164). SEAP levels in culture supernatants were measured at 24 h after stimulation with recombinant human insulin (20 pg/mL). All data are presented as the mean ± SD, n = 3 independent experiments.

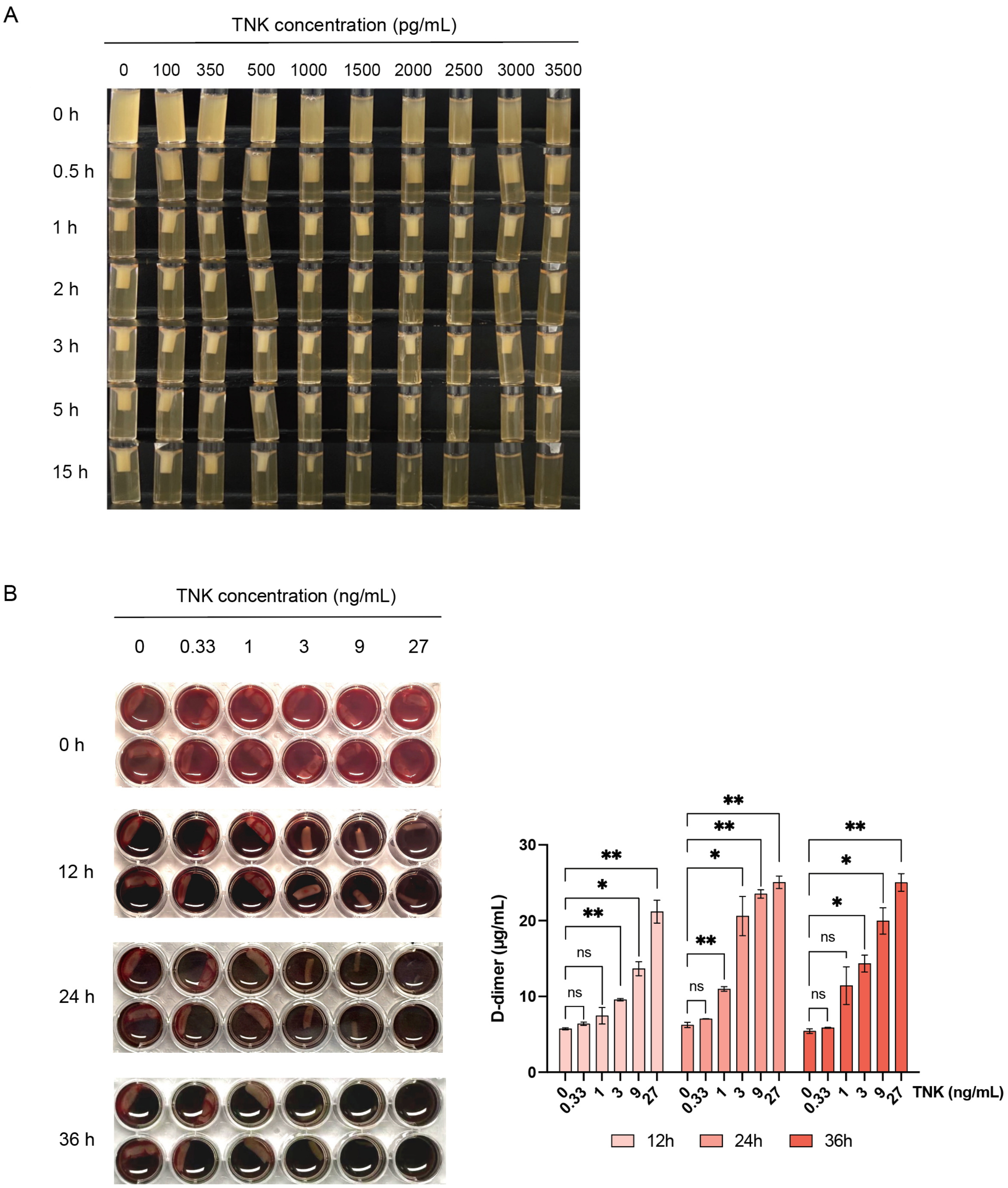

Appendix Fig. S4. Estimation of the TNK efficacy window using *ex vivo* thrombolysis assays. (A) Clot formation prevention assay. Aliquots (20 µL) of conditioned medium of HEK-293 cells transfected with a constitutive TNK expression vector (P_{hCMV}-TNK-pA; pSYQ198) were serially diluted into various concentrations of TNK (0-3.5 ng/mL) together with 20 mM $CaCl_2$ and 0.5 U/mL thrombin in 400 µL platelet-rich plasma (PRP). Clot formation was monitored over 15 h. **(B) Clot lysis standard.** A piece of AMI-like clot produced from platelet-poor-plasma was placed in the whole blood reaction system as described in **Fig. 5E** (500 µL of whole blood and RPMI 1640 medium in 1:1 volumetric ratio in a 24-well plate; n = 2 replicates) supplemented with conditioned medium containing different TNK

concentrations. (left) Clot lysis efficacy was evaluated by photographic analysis after 12 h. (right) Clot lysis was further quantified by measuring the D-dimer level in supernatant.

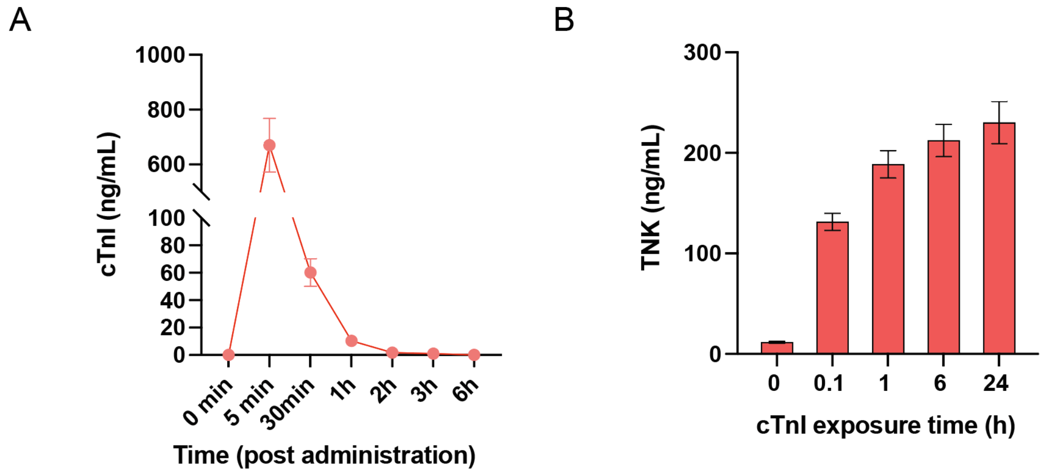

Appendix Fig. S5. CardioProtect sensitivity to short cTnI exposure times. (A) Kinetics of systemic cTnI decay in mice. 80 µg/kg hcTnI was injected into the tail vein of healthy male WT C57BL/6 mice, and blood was taken at different time points for quantification of plasma cTnI levels. Data are presented as the mean ± SEM, n = 4 mice. **(B) Estimation of the cTnI exposure time required for CardioProtect activation.** CardioProtect were incubated in cell culture medium containing 100 ng/mL cTnI for different durations, then the medium was exchanged to cTnI-free DMEM. TNK levels in culture supernatants were quantified at 24 h after medium exchange by ELISA. Data presented are mean ± SD, n = 3 individual experiments.

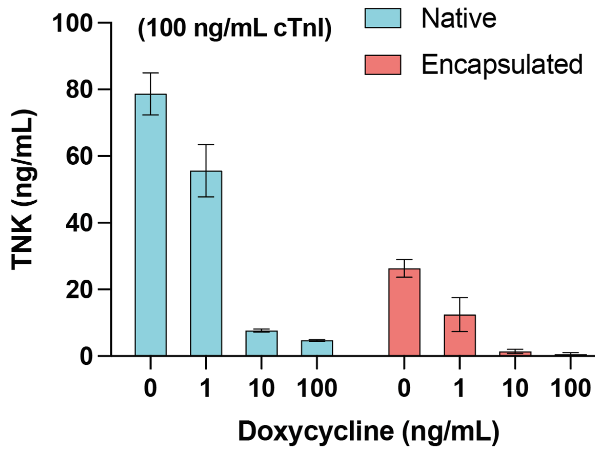

Appendix Fig. S6. Doxycycline-repressible TNK production by encapsulated CardioProtect. 6.5 x10^4 native or encapsulated CardioProtect cells were stimulated with 100 ng/mL cTnI, and cultivated in cell culture medium containing different doses of doxycycline. TNK levels in supernatants were quantified after 24 h by ELISA. Data are presented as the mean ± SEM; n = 3 independent experiments.

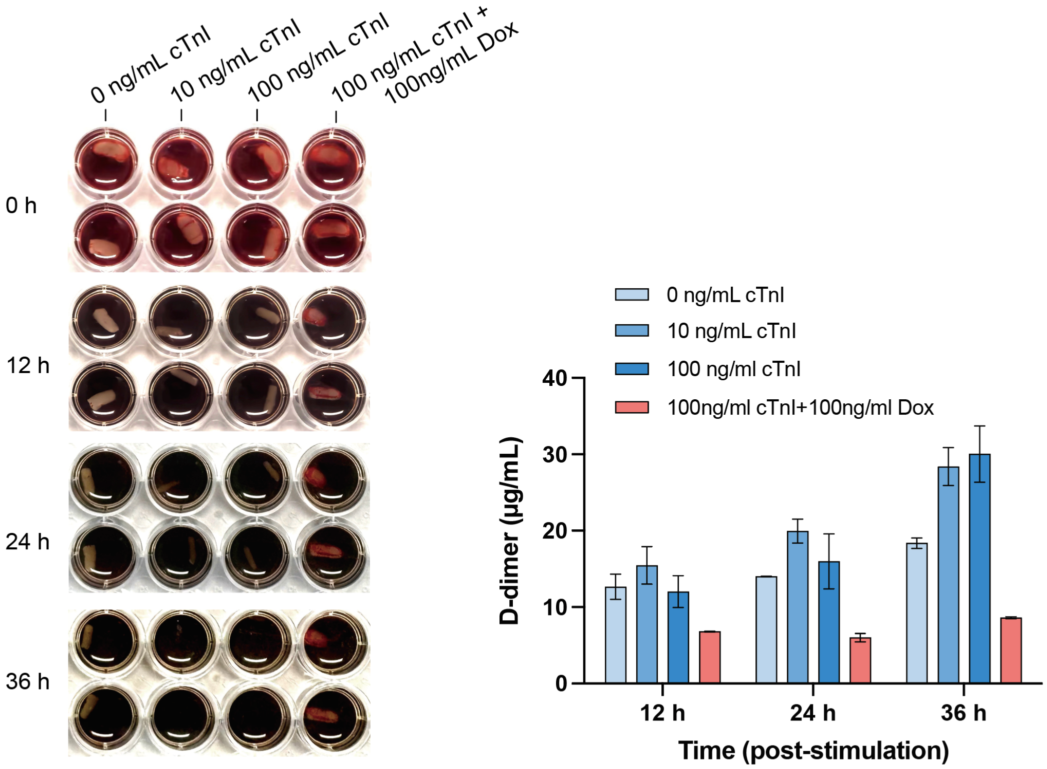

Appendix Fig. S7. Temporal evolution of the *ex vivo* blood culture assay (related to Fig. 5). A piece of AMI-like clot produced from platelet-poor-plasma was placed in the whole blood reaction system as described in **Fig. 5E** (500 µL of whole blood and RPMI 1640 medium in 1:1 volumetric ratio in a 24-well plate; n = 2 replicates) supplemented with conditional medium of encapsulated CardioProtect cells stimulated with different doses of cTnI (0, 10 or 100 ng/mL) and/or doxycycline (0 or 100 ng/mL). The time course of clot lysis was monitored over 36 h by (left) photographic analysis and (right) quantification of supernatant D-dimer levels in each well.

Appendix Table S1. Plasmids designed and used in this study

**Plasmid**	**Description and Cloning Strategy**	**Reference**	**Figures**
pCMV-T7-SB100	Mammalian P_{hCMV}-driven constitutive optimized Sleeping Beauty transposase (SB100X) expression vector.<br>(P_{hCMV}-SB100X-pA)<br>P_{hCMV}: human cytomegalovirus immediate early promoter	Mates et al., 2009	3C, 3D
Mkp37	Mammalian P_{hCMV}-driven constitutive TetR-Elk1 expression vector.<br>(P_{hCMV}-TetR-Elk1-pA)<br>TetR-Elk1: MAPK-inducible *tetO*-specific transcriptional activator	Keeley et al., 2005	3B, 4A, S2, S3
pIR	Mammalian P_{hCMV}-driven constitutive human insulin receptor (IR) expression vector.<br>(P_{hCMV}-IR-pA)	Ye et al., 2017	S3B
pMF111	Tetracycline-responsive TetR-dependent SEAP expression vector.<br>($P_{hCMV*-1}$-SEAP-pA)<br>$P_{hCMV*-1}$: tetracycline-responsive promoter containing a TetR-specific heptameric tetO operator sequence (*tetO*$_7$-$P_{hCMVmin}$);<br>TetR: *Escherichia coli* Tn10-derived tetracycline-dependent repressor of the tetracycline resistance gene;<br>***tetO*:** TetR-specific operator site	Fussenegger et al., 1997	S2B, S3B
pLeo619	Mammalian P_{SV40}-driven constitutive GEMS(RR120)$_{IL-6RBm}$ expression vector.<br>(P_{SV40}-GEMS(RR120)$_{IL-6RBm}$-pA)<br>RR120: azo dye reactive red<br>GEMS(RR120)$_{IL6-RBm}$: RR120-specific GEMS receptor containing a Y759A mutant of interleukin 6 receptor B (IL-6RB) intracellular signal transduction domain (VHH$_{A52}$-GEMS$_{IL-6RBm}$)(Silver & Hunter, 2010)<br>VHH$_{A52:}$ RR120-specific nanobody	Scheller et al., 2018	2B

pLS15	Mammalian P_{hCMV}-driven constitutive STAT3 expression vector.<br>(P_{hCMV}-STAT3-pA)	Schukur et al., 2015	1C, 1D, 1E, 2A, 2B, 2C
pTS566	Mammalian STAT3-specific SEAP expression vector.<br>(O_{STAT3}-$P_{hCMVmin}$-SEAP-pA)<br>O_{STAT3}: STAT3-specific response element<br>$P_{hCMVmin}$: minimal variant of P_{hCMV}	Scheller et al., 2020	1C, 1D, 2A, 2B, 2C
pYL1	Mammalian Ca^{2+}-responsive SEAP expression vector.<br>($(CRE)_3$-$(SRE)_3$-$(NFAT\text{-}IL2)_3$-P_{min*}-SEAP-pA)<br>CRE: cAMP-response element;<br>SRE: serum response element;<br>$NFAT_{IL2}$: nuclear factor of activated T-cells (NFAT)-binding sites of murine IL2 promoter;<br>P_{min}: minimal eukaryotic TATA-box promoter (5'-AGAGGGTATATAATGGAAGCTCGAATTCCAG-3')	Liu et al., 2018	S2B
pLZ284	Mammalian STAT3-specific SEAP expression vector.<br>($(O_{STAT3})_8$-$P_{hCMVmin}$-SEAP-pA)	Shao et al., 2024	S3A
pTS597	Mammalian STAT3-specific expression vector for NanoLuc and TNK.<br>(O_{STAT3}-$P_{hCMVmin}$-NanoLuc-FCS-TNK-pA)<br>FCS: furin cleavage site	Unpublished	1E
pIL-6R	Mammalian P_{hCMV}-driven constitutive human interleukin 6 receptor (NCBI ID: NC_000001.11) (IL-6R) expression vector.<br>(P_{hCMV}-IL-6R-pA)	This work	S3A
pLeo1058	Mammalian P_{SV40}-driven constitutive expression vector for A2 (anti cTnI scFv(Fukunaga & Tsumoto, 2013) fused to the GEMS receptor scaffold.<br>(P_{SV40}-SP-A2-$GEMS_{IL\text{-}6RBm}$-pA)<br>SP: secretory signal peptide<br>$GEMS_{IL\text{-}6RBm}$: synthetic F93A mutant of EpoR fused to a Y759A mutant of IL-6RB intracellular signal	This work	1C

	transduction domain; $GEMS_{FGFR1\_int}$, synthetic F93A mutant of EpoR fused to $FGFR1_{int}$		
pLeo1059	Mammalian P_{SV40}-driven constitutive expression vector for 2B12 (anti cTnI scFv(Conroy et al, 2012)) fused to the GEMS receptor scaffold.<br>(P_{SV40}-SP-2B12-$GEMS_{IL-6RBm}$-pA)	This work	1C
pLeo1060	Mammalian P_{SV40}-driven constitutive $TropR_{IL6-RBm}$ expression vector.<br>(P_{SV40}-$TropR_{IL6-RBm}$-pA)<br>$TropR_{IL6-RBm}$: cTnI-specific homodimeric GEMS receptor containing IL6-derived intracellular signal transduction domains (SP-2B12-A2-$GEMS_{IL-6RBm}$)	This work	1C, 1D, 1E, 2A, 2B, 2C
pLeo1061	Mammalian P_{SV40}-driven constitutive $TropR_{FGFR1}$ expression vector.<br>(P_{SV40}-$TropR_{FGFR1}$-pA)<br>$TropR_{FGFR1}$: cTnI-specific homodimeric GEMS receptor containing FGFR1-derived intracellular signal transduction domains (SP-2B12-A2-$GEMS_{FGFR1\_int}$)	This work	3B, S2B
pLeo1120	Mammalian P_{hPGK}-driven constitutive $TropR_{IL6-RBm}$ expression vector.<br>(P_{hPGK}-$TropR_{IL6-RBm}$-pA)	This work	2B
pLeo1164	Mammalian P_{SV40}-driven constitutive $TropR_{FGFR2b}$ expression vector.<br>(P_{SV40}-$TropR_{FGFR2b}$-pA)<br>$TropR_{FGFR2b}$: cTnI-specific homodimeric GEMS receptor containing FGFR2b-derived intracellular signal transduction domains (SP-2B12-A2-$GEMS_{FGFR2b\_int}$)	This work	3B, S2B, S3B
pLeo1165	Mammalian P_{SV40}-driven constitutive $TropR_{EGFR}$ expression vector.<br>(P_{SV40}-$TropR_{EGFR}$-pA)<br>$TropR_{EGFR}$: cTnI-specific homodimeric GEMS receptor containing EGFR-derived intracellular signal transduction domains (SP-2B12-A2-$GEMS_{EGFR\_int}$)	This work	3B, S2B
pLeo1171	Mammalian P_{SV40}-driven constitutive $TropR_{VEGFR2}$ expression vector.	This work	S2

	(P_{SV40}-TropR$_{VEGFR2}$-pA)<br>TropR$_{VEGFR2}$: cTnI-specific homodimeric GEMS receptor containing VEGFR-derived intracellular signal transduction domains (SP-2B12-A2-GEMS$_{VEGFR2_int}$)		
pSYQ198	Mammalian P_{hCMV}-driven constitutive TNK expression vector.<br>(P_{hCMV}-TNK-pA)	This work	4A, 4C
pSYQ267	Mammalian TetR-specific TNK expression vector.<br>($P_{hCMV*-1}$-TNK-pA)	This work	4A-4G
pSYQ294	Mammalian TetR-specific expression vector for NanoLuc and TNK.<br>($P_{hCMV*-1}$-NanoLuc-P2A-TNK-pA)<br>P2A: porcine teschovirus-derived ribosome skipping sequence optimized for bicistronic expression in mammalian cells	This work	4A
pSYQ326	Mammalian TetR-specific expression vector for NanoLuc and TNK.<br>($P_{hCMV*-1}$-TNK-P2A-NanoLuc-pA)	This work	4A
pSYQ367	SB100X-specific transposon containing a TetR-specific TNK expression vector.<br>(ITR$_{SB}$-$P_{hCMV*-1}$-TNK-pA-ITR$_{SB}$)<br>ITR$_{SB}$: inverted terminal repeats of Sleeping Beauty transposase.	This work	Fig. 5
pSYQ377	Mammalian TetR-specific doxycycline-responsive NLuc expression vector.<br>($P_{hCMV*-1}$-NLuc-pA)	This work	3B, S2A
pYX415	Mammalian calcium-responsive NLuc expression vector containing three NFAT$_{IL4}$-specific response elements.<br>((NFAT$_{IL4}$)$_{10}$-P_{min*}-NLuc-pA)	This work	S2
pYX941	Mammalian P_{SV40}-driven constitutive expression vector for A2 fused to the GEMS receptor scaffold containing a C-terminal EGFP tag.<br>(P_{SV40}-SP-A2-GEMS$_{IL-6RBm}$-EGFP-pA)	This work	S1

pYX942	Mammalian P_{SV40}-driven constitutive expression vector for 2B12 fused to the GEMS receptor scaffold containing a C-terminal EGFP tag.<br><br>(P_{SV40}-SP-2B12-$GEMS_{IL\text{-}6RBm}$-EGFP-pA)	This work	S1
pYX943	Mammalian P_{SV40}-driven constitutive expression vector for EGFP-tagged $TropR_{IL6\text{-}RBm}$.<br><br>(P_{SV40}-$TropR_{IL6\text{-}RBm}$-EGFP-pA)	This work	S1
pBS804	SB100X-specific transposon containing constitutive expression units for $TropR_{FGFR2b}$, modified red fluorescent protein (mRuby2) and gene conferring zeocin resistance (ZeoR).<br><br>(ITR_{SB}-P_{SV40}-$Trop_{FGFR2b}$-pA::P_{RPBSA}-mRuby2-P2A-ZeoR-pA-ITR_{SB})<br>P_{RPBSA}: synthetic constitutive promoter	This work	3C-3F
pBS878	SB100X-specific transposon containing a TetR-specific NanoLuc expression unit and a constitutive expression unit for yellow fluorescent protein (YPet) and gene conferring puromycin resistance (PuroR).<br><br>(ITR_{SB}-$P_{hCMV*\text{-}1}$-NanoLuc-pA::P_{RPBSA}-YPet-P2A-PuroR-pA-ITR_{SB})	This work	3C-3F
pBS880	SB100X-specific transposon containing constitutive expression units for TetR-Elk1, blue fluorescent protein (BFP) and gene conferring blasticidin resistance (BlastR).<br><br>(ITR_{SB}-P_{hCMV}-TetR-Elk1-pA::P_{RPBSA}-BFP-P2A-BlastR-pA-ITR_{SB})	This work	3C-3F

Abhinand CS, Raju R, Soumya SJ, Arya PS & Sudhakaran PR (2016) VEGF-A/VEGFR2 signaling network in endothelial cells relevant to angiogenesis. *J Cell Commun Signal* 10: 347–354

Conroy PJ, O'Kennedy RJ & Hearty S (2012) Cardiac troponin I: a case study in rational antibody design for human diagnostics. *Protein Engineering, Design and Selection* 25: 295–305

Fukunaga A & Tsumoto K (2013) Improving the affinity of an antibody for its antigen via long-range electrostatic interactions. *Protein Engineering Design and Selection* 26: 773–780

Greulich H, Chen T-H, Feng W, Jänne PA, Alvarez JV, Zappaterra M, Bulmer SE, Frank DA, Hahn WC, Sellers WR, *et al* (2005) Oncogenic transformation by inhibitor-sensitive and -resistant EGFR mutants. *PLoS Med* 2: e313

Liao RG, Jung J, Tchaicha J, Wilkerson MD, Sivachenko A, Beauchamp EM, Liu Q, Pugh TJ, Pedamallu CS, Hayes DN, *et al* (2013) Inhibitor-sensitive FGFR2 and FGFR3 mutations in lung squamous cell carcinoma. *Cancer Res* 73: 5195–5205

Reichhart E, Ingles-Prieto A, Tichy A-M, McKenzie C & Janovjak H (2016) A Phytochrome Sensory Domain Permits Receptor Activation by Red Light. *Angew Chem Int Ed Engl* 55: 6339–6342

Silver JS & Hunter CA (2010) gp130 at the nexus of inflammation, autoimmunity, and cancer. *J Leukoc Biol* 88: 1145–1156

Simonsen JL, Rosada C, Serakinci N, Justesen J, Stenderup K, Rattan SIS, Jensen TG & Kassem M (2002) Telomerase expression extends the proliferative life-span and maintains the osteogenic potential of human bone marrow stromal cells. *Nat Biotechnol* 20: 592–596
